# Investigating midwives’ barriers and facilitators to multiple health promotion practice behaviours: a qualitative study using the theoretical domains framework

**DOI:** 10.1186/s13012-019-0913-3

**Published:** 2019-06-18

**Authors:** Julie M. McLellan, Ronan E. O’Carroll, Helen Cheyne, Stephan U. Dombrowski

**Affiliations:** 1 Division of Psychology, Faculty of Natural Sciences, University of Stirling, Stirling, Scotland UK; 2 Nursing Midwifery and Allied Health Professions Research Unit, University of Stirling, Stirling, Scotland UK; 3 Faculty of Kinesiology, University of New Brunswick, Bathurst, Canada

**Keywords:** Midwives, Health promotion, Multiple health behaviours, Theoretical domains framework

## Abstract

**Background:**

In addition to their more traditional clinical role, midwives are expected to perform various health promotion practice behaviours (HePPBes) such as informing pregnant women about the benefits of physical activity during pregnancy and asking women about their alcohol consumption. There is evidence to suggest several barriers exist to performing HePPBes. The aim of the study was to investigate the barriers and facilitators midwives perceive to undertaking HePPBes.

**Methods:**

The research compromised of two studies.

Study 1: midwives based in a community setting (*N* = 11) took part in semi-structured interviews underpinned by the theoretical domains framework (TDF). Interviews were analysed using a direct content analysis approach to identify important barriers or facilitators to undertaking HePPBes.

Study 2: midwives (*N* = 505) completed an online questionnaire assessing views on their HePPBes including free text responses (*n* = 61) which were coded into TDF domains. Study 2 confirmed and supplemented the barriers and facilitators identified in study 1.

**Results:**

Midwives’ perceived a multitude of barriers and facilitators to carrying out HePPBes. Key barriers were requirements to perform an increasing amount of HePPBes on top of existing clinical work load, midwives’ cognitive resources, the quality of relationships with pregnant women, a lack of continuity of care and difficulty accessing appropriate training. Key facilitators included midwives’ motivation to support pregnant women to address their health. Study 1 highlighted strategies that midwives use to overcome the barriers they face in carrying out their HePPBes.

**Conclusions:**

Despite high levels of motivation to carry out their health promotion practice, midwives perceive numerous barriers to carrying out these tasks in a timely and effective manner. Interventions that support midwives by addressing key barriers and facilitators to help pregnant women address their health behaviours are urgently needed.

**Electronic supplementary material:**

The online version of this article (10.1186/s13012-019-0913-3) contains supplementary material, which is available to authorized users.

Contributions to the literature

- This research systematically examines barriers and facilitators midwives perceive in helping pregnant women with multiple health behaviour change
- The theoretical domains framework is used to understand midwives’ multiple health promotion practice behaviours across a range of health topics
- The barriers and facilitators health care professionals face in addressing multiple health behaviour change topics will help inform interventions to support the uptake of evidence-based guidelines into routine clinical healthcare practice

## Introduction

In many developed countries, the public health focus for midwives has extended from health protection issues, such as reducing maternal and infant mortality and preventing the spread of disease, to health promotion topics, such as smoking cessation, and weight management [1]. In the United Kingdom (UK), midwives are expected to perform multiple health promotion practice behaviours (HePPBes) for a variety of health promotion topics throughout pregnancy and postnatally. Examples of HePPBes include monitoring carbon monoxide levels, discussing recommended daily fruit and vegetable intake or delivering an alcohol brief intervention (in the UK, the booking appointment takes place between 8 and 12 weeks gestation and is the first routine antenatal appointment).

HePPBes are outlined in the various policies, strategies and guidelines published by government and public-sector bodies, which either directly or indirectly implicate midwives as public health professionals [2–4]. For example, in the UK, the National Institute of Clinical Excellence (NICE) Smoking: stopping in pregnancy and after childbirth guidelines outline that midwives participate in up to 12 different smoking cessation-related HePPBes during pregnancy, such as measuring carbon monoxide levels, asking the woman if they or anyone in their household smokes and referring to NHS Stop smoking services [4]. Whilst the NICE Weight management before, during and after pregnancy guidelines [5] outline various HePPBes including measuring weight and height, asking questions about the pregnant women’s diet and physical activity and giving dietary and physical activity advice. For pregnant women with a BMI ≥ 30, midwives are expected to carry out additional HePPBes such as offering referral to a dietitian. Considering the variety of health promotion topics to be addressed during pregnancy, midwives face a high health promotion workload [6–10].

The factors related to midwives performing multiple HePPBes are poorly understood. Previous studies have examined maternal health care professionals’ behaviour using the theoretical domains framework [TDF; 11 [11]]. However, these studies examined single health-risk topic such as smoking cessation [12], weight management and obesity [13] and physical activity [14]. The TDF provides a comprehensive grouping of the overlapping constructs within behavioural theories. The original version (TDF v1) summarises the main factors of relevant behaviour change theories into 12 independent domains [11]. The TDF v1 has been validated through the development of a refined version (TDF v2; [15]).

Midwives experience several challenges in undertaking multiple HePPBes such as a shortage of resources [6], a lack of clarity about their public health role [7, 8] and lack of self-efficacy [8, 9]. However, limited evidence exists on the barriers and facilitators midwives perceive in undertaking multiple HePPBes. This study applies a theoretical approach to investigate potentially relevant factors at a multiple behaviour level.

### Research aim

The aim of this study is to investigate midwives’ barriers and facilitators to performing multiple HePPBes across various health promotion topics using the theoretical domains framework in qualitative interviews (study 1) and free text questionnaire responses (study 2).

## Methods

This study reports two different sources of qualitative data gathered through interviews and questionnaires. Interviews obtained detailed evidence about the barriers and facilitators midwives experience in carrying out their HePPBes. The questionnaires used an open-ended question to capture additional comments on barriers and facilitators that midwives may have had about their HePPBes.

### Study 1

#### Study design

Qualitative semi-structured interview study.

#### Participants

Midwives working in a community setting were eligible to participate if they were qualified, practising midwives employed by an NHS health board in central Scotland. Recruitment involved JM, a researcher previously unknown to participants, visiting an out-patient maternity clinic and providing 12 midwives with information about the study. The information provided to midwives included the reason for carrying out the research to inform JM’s PhD to develop an intervention to support midwives in addressing health behaviours with pregnant women. Eleven midwives agreed to take part. One midwife opted not to take part in the study.

#### Interview topic guide

The interview topic guide (see Additional file 1) contained (i) demographic questions (number of years of experience and job title) and (ii) questions based on each of the 12 TDF (v1) domains [11]. The behavioural category of interest, within the topic guide, was specified as: “supporting pregnant women to change their health behaviour” and the questions were designed to elicit beliefs about the behaviour in relation to each domain.

To remind midwives of the target behaviour of interest, an A4 prompt card was placed in front of them outlining typical examples of women’s health behaviours to be addressed (see the prompt card in Additional file 2). The behaviour was specified using terms Target, Action, Context and Time, known as the TACT principle [16]. TACT summarises the behaviour in terms of doing what, to whom, in a given context and at a specific time [17]. The behaviour was specified as: “All the things you do in a routine antenatal care consultation, including asking questions, to support pregnant woman change their health behaviours”. The TACT specification complements the general TDF definition used within the topic guide by breaking down of what was meant by “supporting pregnant women to change their health behaviour”.

#### Procedure

Face-to-face semi-structured interviews were conducted by JM (a female PhD researcher and Health Psychologist with previous experience of supporting midwives’ behaviour change practice) on two separate occasions in October 2016. Interviews took place within consultation rooms at an out-patient maternity clinic in central Scotland. Information about the study was provided verbally and in written format. Interviews lasted between 27 and 76 min (mean ± SD, 43 ± 14). All interviews were audio recorded and anonymously transcribed verbatim. The demographic data was entered into a Microsoft Excel spread sheet. The consolidated criteria for reporting qualitative research (COREQ; [18]) was used to ensure all aspects of the qualitative research had been reported (a copy of the checklist is provided in Additional file 3).

#### Analysis

Transcripts were stored as Microsoft Word documents. Qualitative data analysis was based on recommendations for conducting TDF based qualitative research [19] and involved the following ten steps:

1. Interviews were read several times by JM to ensure familiarity with the data.
2. One interview was jointly coded by JM and SD to develop a coding strategy.
3. Two interviews were coded by JM using a directed content analysis approach [20] in which interview content was placed in the most relevant TDF domain(s). Responses which could be attributed to more than one domain were coded into multiple domains.
4. The coding of the two interviews was checked by SD. Where discrepancies in coding occurred, discussion took place to reach a consensus.
5. The remaining interviews were coded by JM.
6. Data saturation was reached as the final three transcripts did not introduce any additional barriers and facilitators than those already identified.
7. Summaries of domain codings were produced by JM and checked by SD.
8. Identification of relevant theoretical domains was identified by consensus discussion between JM & SD. Relevance of a domain was based on the following criteria: (i) high frequency of specific beliefs and/or (ii) existence of conflicting beliefs and/or (iii) indication of clear beliefs that may influence the behaviour of interest [21].
9. Views were generated for relevant domains by JM and coded as being either generic (views which are made in reference to HePPBes in general) or behaviour specific (views which are in reference to a specific health promotion behaviour).
10. The views generated were checked by HC (a Professor of Midwifery) to ensure they made sense from a midwifery perspective.

#### Ethical approval

The University of Stirling Psychology Ethics Committee approved the study. NHS Research and Development approval was granted by Greater Glasgow and Clyde Health Board (R&D reference: GN16OG406).

### Study 2

#### Study design

Online questionnaire study including a qualitative open-ended question.

#### Participants

Individuals registered as a qualified midwife or training to be a midwife, worldwide, were eligible to take part. Recruitment took place online between the February and May 2018. Advertisements were placed on discussion forums, email lists and social media pages. The study was endorsed by the Royal College of Midwives on their Facebook and Twitter pages. Advertisements contained an URL link to the online study platform Qualtrics where the questionnaire was hosted. Overall, 719 participants consented to take part in the study and confirmed they were either a qualified or student midwife. Of those, 214 completed less than 95% of the questionnaire and therefore were excluded from further analysis. Complete responses were obtained from 505 participants.

#### Questionnaire

The questionnaire examined factors relevant to HePPBes. At the end of the questionnaire, participants were asked: “If you have any other comments on your Public Health role then please include them below”. The current paper reports on the qualitative data obtained from this question.

#### Procedure

Midwives accessed the questionnaire by clicking on the URL contained within the online advertisement. Following presentation of study information and eligibility criteria, consent was obtained by the midwife selecting an electronic check box. A screening question: “Are you a qualified or student midwife?” was presented as a method of reducing the likelihood of non-midwives completing the questionnaire. If the response was “no”, then participants were thanked for their interest in the study and exited from the questionnaire. At the end of the questionnaire, midwives were offered the opportunity to be entered into a prize draw to win 1 of 4x £25 shopping vouchers.

#### Analysis

Analysis of the qualitative questionnaire data involved the following five steps:

1. Responses were read several times by JM to ensure familiarity with the data.
2. Responses were coded by JM using a directed content analysis approach [20] in which responses were placed in the most relevant TDF domain. If a response could be coded into more than one domain, a decision was made by JM as to the most appropriate domain.
3. Coding was checked by SD.
4. The number of responses coded into each domain was calculated by JM.
5. JM checked how much the barriers identified reflected those in study 1 and if there were any additional barriers or facilitators identified.

#### Ethical approval

The University of Stirling’s General University Ethics Panel approved the study (GUEP316).

## Results

### Study 1

#### Participants

All 11 participants were female, employed as community midwives, except one who worked as a Senior Charge Midwife. The mean number of years of experience as a qualified midwife was 22 (range from 3 to 31).

#### Reviewing of coding

Agreement between coders for two interviews was 76% and 88% for the first and second interview respectively, and disagreement for the same interviews was 17% and 5% respectively. The mean agreement was 82% and mean disagreement was 11%. An additional 7% of codes were suggested by the second coder for each interview.

#### Relevant theoretical domains

All barriers and facilitators could be identified within the TDF. Nine of the 12 TDF domains were classified as important in understanding the barriers (*b* = barrier) and facilitators (*f* = facilitator) to undertaking HePPBes. Table 1 lists these domains alongside a domain descriptor.

**Table 1:** Criteria for why TDF domains were identified as key in understanding the barriers and facilitators midwives experience in undertaking multiple HePPBes

TDF domain	Domain description	(i) High frequency of specific beliefs	(ii) Existence of conflicting beliefs	(iii) Indication of clear beliefs
Professional role and identity	Views of how HePPBes relate to the professional role of being a midwife	✓	✓	
Beliefs about consequences	Expectations about what would occur if midwives perform HePPBes		✓	✓
Motivation and goals	Reasons for carrying out or not carrying out HePPBes	✓		
Memory/Attention and decision processes	The ability to remember, observe and select in relation to HePPBes	✓		✓
Environmental context and resources	The effects of the healthcare setting on HePPBes and the impact of what is available to midwives (in terms of physical and psychological resources) on HePPBes			✓
Social influences	The interpersonal processes which influence midwives’ cognitions, emotions and HePPBes	✓	✓	
Emotion	Feelings about performing HePPBes			✓
Behavioural regulation	Midwives’ attempts to influence HePPBes	✓		
Nature of the behaviour	Midwives’ descriptions of how they have carried out HePPBes in the past and how HePPBes operate within the NHS	✓		

The identified domains are outlined below and a table containing the associated belief statements are provided in Additional file 4.

### Professional role and identity

Midwives mostly saw HePPBes as part of their professional role (f): “I just see it as my job” (M10) and “I think public health is an essential part our role” (M7). However, some thought that several HePPBes could be addressed prior to conception, especially around weight management (b): “She’s thirty-five and she’s pregnant, so why is it suddenly the midwife that has to look into that?” (M3). Midwives frequently mentioned that the role of the midwife had evolved from providing traditional midwifery care (e.g. measuring the growth of the baby) to having a strong focus on undertaking HePPBes (b): “They seem to keep adding to the list of things we’re expected to do”(M11), and some midwives expressed a feeling that their traditional professional role was being eroded (b): “Our role now, as community midwives, seems to be for referring on … it feels as if your role’s been kind of eroded at” (M10).

### Beliefs about consequences

Midwives mentioned several consequences that potentially impact their HePPBes. Contrasting beliefs about how HePPBes impacted on the relationship with the woman were voiced. If performed well, midwives believed it could be useful in gathering information about aspects of the women’s wellbeing (f). However, some stated that performing HePPBes could potentially damage the relationship if they were not carried out carefully, particularly for HePPBes related to weight management (b): “Women get quite offended at that one” (M10).

Similarly, contrasting beliefs about the womens’ receptiveness to HePPBes emerged. Some midwives reported that women expect them to carry out HePPBes (f): “Most women are quite receptive to that because they know they’re pregnant and know it’s not just about their health anymore” (M11). Other midwives said that women were not receptive to HePPBes (b): “It seems to be that everything is piled on to this booking visit and I don’t think it’s fair on the women either” (M3).

The time it takes to perform HePPBes was seen as a clear barrier with appointments over running the allotted time which could impact on other women (b): “You run over and then people are kept waiting.” (M11). Furthermore, midwives held a clear belief that HePPBes had the potential to have positive health benefits for the women and their child (f): “Absolutely, there’s a huge knock-on effect” (M5). Clear views on the short-term impact of HePPBes depended on the behavioural topic. For instance, smoking was perceived as an issue that could be dealt with during pregnancy (f): “This is probably a time, particularly for the smokers, they’ve got that motivation for the baby to change” (M5). Meanwhile, the impact of diet-related HePPBes was considered as unobservable (b): “I’m never going to know whether she’s changed her diet, or even if she did change her diet, whether that’s going to last” (M6). Some midwives expressed a clear belief that it was rewarding for them to observe the benefits of women engaging in health behaviour change attributed to their HePPBes (f): “That is rewarding if you feel like you’ve helped someone make a change in their life.” (M11). Benefits in reducing future workload if HePPBes were carried out effectively were noted (f): “If we do our job well at the booking clinic and women take that on board then we don’t have as much to do” (M2).

### Motivation and goals

Midwives frequently reported being highly motivated to undertaking HePPBes to benefit the long-term health of the woman and the baby (f): “I think it’s a huge window of opportunity for midwives” (M5). However, HePPBes were not a priority if there were conflicting clinical risks to the woman and/or baby such as patient safety or adult/child protection issues (b): “I’d say it’s definitely secondary though, obviously check the woman’s blood pressure, making sure she’s well, doing urine analysis, making sure there’s no infections, ruling out pre-eclampsia, listening to baby. That comes first and everything else, I think, would come second to that.” (M11).

### Memory/attention and decision processes

Midwives described being prompted by the woman’s maternity notes to cover all HePPB topics (f): “My booking visit would be just going through that book with them because everything I need to tell them is in there, it’s a good thing for me cause it saves me forgetting to stop to talk about things” (M3) which also acted as a prompt to HePPBes at follow-up appointments (f): “I usually always have a wee flick through the notes at the beginning just to check if there’s any kind of outstanding issues to be aware of (M11)”.

If the woman wanted to discuss a particular behaviour, midwives prioritised this (f): “If the woman is worried about her weight, I’m happy to talk about it at every appointment, but if she’s not then I’m not gonna bring it up”, (M6). Some midwives covered a topic in depth if they felt it was of specific relevance (f): “Say I did three bookings yesterday one of them would have had none of these problems, one of them had a BMI was over 35 so that’s the one I concentrated on.” (M5).

Intuition was frequently reported as guiding decision making in relation to HePPBes (f): “If I get vibes from them, that actually they do know” (M5) and “I just have to go with my gut at the time” (M6)*.* Midwives also based performing HePPBes on the physical health of the woman during the appointment (b): “If they are very sick or they’ve had bleeding, then I’ll just say, ‘we’ll talk about this another time’ because it’s not appropriate to get ahead of ourselves” (M2).

### Environmental context and resources

Changes in health care service provision (e.g. changes in timing of booking appointments) were perceived as making it more difficult to carry out HePPBes (b): “… with continuity of care being removed from us we’re not getting the same chance to see the same women again so I find it a bit harder to address things.” (M10).

Some midwives held a belief that accessibility to resources such as training related to HePPB could be improved (b): “It’s quite haphazard how you can get on to these things” (M4). Materials related to HePPBes were generally perceived as high quality (f): “‘Ready Steady Baby’ is I think a fantastic book” (M10). However, some felt the wording of questions within maternity notes made them difficult to ask (b): “That’s a barrier to me asking, because I actually don’t ask the way it’s worded on that because it doesn’t make sense.” (M4). A belief that there were too many HePPBes to undertake in too little time was apparent (b): “We’ve also got to try and work within the time constraints” (M9). Some midwives believed that the woman’s health status at the booking appointment affected the degree to which they could carry out HePPBes (b): “The booking appointment is really difficult for some women to sit there and actually not vomit” (M7). Physical cues were mentioned as prompts to undertake HePPBes (f): “If you pick up a book and it stinks of smoke, you know, you might well say, how you getting on?” (M2).

### Social influences

Women were reported as a strong influence on midwives HePPBes and were seen to increasingly inform themselves through online sources. This was perceived as helpful to recommend high-quality information (f): “Get them to use websites because most of them are on computer all the time anyway” (M3) and unhelpful due to the potential to increase stress (b): “A lot of the women have got health anxieties and that’s fuelled by the internet” (M2). Mixed views emerged about how accurately women reported some health behaviours such as alcohol consumption, which impacted on health promotion efforts. Some midwives perceiving accurate accounts (f): and others reporting the opposite (b): “Alcohol, I think, is probably one that’s probably hidden, getting women to be honest is probably very difficult” (M10).

Team working and social support was seen as helpful in resolving issues regarding HePPBes (f): “My kind of closest colleagues, we’d probably have a wee chat and we’ll probably complain about how we’re meant to put this in amongst everything else that people want out of us.” (M10). Intergroup conflict was perceived by some in relation to performing HePPBes (b): “It’s come up in the tearoom and there will be conversations with people saying, ‘Oh public health that’s a load of nonsense’ and I’ll sit there quite openly and say ‘I think it’s one of the best things that’s ever occurred’” (M7).

Midwives described shifting social and group norms useful to normalise addressing health behaviours (f): “There’s very few people that are not happy to answer these questions nowadays because we’ve been doing this for so long they expect it and they do all talk amongst each other” (M7). However, social norms appeared to be unhelpful in normalising obesity (b) “If a lady’s got a BMI of not over 30, I still sort of don’t see it as a huge issue with them” (M7).

Some saw a midwife’s own body mass index (BMI) potentially making it harder to perform weight management HePPBes (b): “I think midwives find it really difficult because if you’re big yourself they’re looking at you thinking: ‘well, she’s got a cheek’, if you’re small they’re looking at you thinking: ‘you have never had a problem in your life’” (M10).

### Emotion

Carrying out HePPBes was associated with a range of positive emotions if these were seen to result in positive outcomes (f): “You feel dead pleased they actually brought it up again” (M9). Some reported concerns about performing specific HePPBes (b): “I do find it causes me anxiety if I know I’m going to tell her today that we’re doing a Social Work referral.” (M10). Carrying out HePPBes was potentially stressful (b): “Sometimes I’m thinking you just want to do the right thing, which is hard sometimes” (M5) and draining (b): “I’m exhausted after a clinic because you feel as if you want to have your senses hyper alert” (M9).

### Behavioural regulation

Midwives described using behavioural regulation strategies such as using maternity notes as a prompt to cover all HePPBes, writing notes in SWHMMR as prompt for carrying out HePPBes follow-up appointments, carrying out HePPBes whilst performing clinical tasks, e.g. asking questions about physical activity while taking bloods (f): “I have to say I multi task. I’ll be testing the urine while I’m asking about how they feel in pregnancy and had they had any sickness and how they’re getting on with eating.” (M7). For a list of strategies reported, see Additional file 5.

### Nature of the behaviours

The majority of HePPBes took place at the booking appointment when there is usually the most time to undertake HePPBes (f). Midwives reported HePPBes as being routine practice (f): “We’ve got to tick boxes, we’ve got to tick that we’ve discussed alcohol, we’ve discussed smoking” (M10). The habitual nature of performing HePPBes included the strategies used to regulate health promotion practice as well as the behaviours themselves.

### Study 2 results

#### Participants

Forty-seven fully qualified midwives and 14 student midwives provided a statement to the final question. The majority (92%) were based in the UK. The mean number of years of experience as a qualified midwife was 17 (range from 1 month to 40 years).

#### Relevant theoretical domains

Responses were coded into seven TDF domains: professional role and identity, beliefs about consequences, motivation and goals, environmental context and resources, social influences, emotion and beliefs about capabilities. The definitions for each domain are the same as those presented in study 1. The domains are presented in terms of (i) the number of responses and (ii) supporting evidence.

#### Environmental context and resources

Twenty-six responses were coded as environmental context and resources focusing on a need for improved resources, particularly a need for more time, wider access to online materials: “Apps and online mediums for encouraging behaviour change may take the pressure off midwives” and more accessibility to training*.* Some responses stressed the need for continuity of care.

#### Beliefs about consequences

Nine responses were coded as beliefs about consequences. The potential for weight management HePPBes to impact the midwife-woman relationship was mentioned. Mixed responses about women’s receptiveness to HePPBes emerged*.*

#### Motivation and goals

Nine motivation and goals responses suggested high levels of motivation to carry out HePPBes*.* Some midwives indicated that the degree to which they were able to support women was not ideal.

#### Social influences

Eight responses were coded as social influences and focused on midwives’ own health status in relation to undertaking HePPBes. Some midwives described their own health behaviours and status helping or hindering HePPBes: “My own lifestyle and motivation in public health topics can impact the delivery and communication when approaching topics with women”*.* Others reported that their health status was irrelevant: “Don’t confuse my welfare with those of the woman and baby I’m caring for... public health roles should not be judged by the delivering midwife”.

#### Professional role and identity

Three responses were coded as professional role and identity commenting on a need for health promotion topics to be tackled before pregnancy and the demands placed on midwives to fulfil multiple professional roles.

#### Emotion

Three responses coded as emotion focused on the taxing nature of the job and the potential negative health consequences of burn-out.

#### Beliefs about capabilities

Three responses coded as beliefs about capabilities highlighted that midwives potentially feel more confident in addressing health promotion topics which have greater attention placed on them in health policy and that capability to undertake HePPBes was reliant on resources such as training and time*.*

#### Integration of study 1 and 2 findings

Table 2 presents the integration of the findings from both studies by highlighting whether the views demonstrated in study 1 were supported by the responses generated in study 2. The table shows that six of the nine domains identified as important in study 1 were supported by responses from study 2.

**Table 2:** Evidence of midwives’ views identified in study 1 also present in the study 2 responses

Key TDF domains from study 1	Study 2 supports or extends study 1 findings (✓ = yes or ✘ = no evidence)	Details of how study 2 responses relate to study 1 barriers and facilitators
Professional role and identity	✓	Study 2 responses support those in study 1 that suggest midwives are expected to address various topics that could be targeted prior to pregnancy (b). However, unlike the study 1 findings which suggested that other health professionals could potentially address some health promotion topics prior to pregnancy, there was a suggestion in the study 2 responses that midwives could be the professional to do this (e.g. by visiting schools). There was also further evidence of the perception that the role of the midwife has evolved to incorporate a wide variety of HePPBes (b).
Beliefs about consequences	✓	Study 2 responses strengthen the findings of study 1 which suggested that midwives believed HePPBes related to weight management were most likely to have a negative impact on the midwife-woman relationship (b). Study 2 also provides further evidence of the differing beliefs that midwives have regarding how receptive women are to HePPBes (b&f).
Motivation and goals	✓	Study 2 responses support those of study 1 which demonstrated that midwives are motivated to carry out their health promotion practice (f) but competing clinical demands mean that it was a secondary goal (b).
Memory, attention and decision processes	✘	No further evidence identified.
Environmental context and resources	✓	The responses from study 2 support the findings of study 1 which outlined issues including not having enough time to address health promotion meaningfully (b), problems accessing training (b) and a lack of continuity of care (b) in influencing midwives’ HePPBes. Study 2 also identified the need for greater access to online materials which was not reported in study 1.
Social influences	✓	The findings of study 1 suggested that some midwives believed their own health status, specifically their BMI, could influence their health promotion practice by exerting social pressure. However, the responses generated by study 2 show that there is widely differing regard as to whether midwives feel their own health status has a potential impact on their health promotion practice (b&f).
Emotion	✓	Study 2 responses supported the study 1 finding that midwives’ HePPBes could potentially be influenced by the exhaustive nature of the midwifery role (b). However, study 1 did not identify the potential impact of burn-out on midwives’ own health as was suggested by the study 2 responses. This is perhaps as study 1 contained purely midwives working in a community setting only.
Behavioural regulation	✘	No further evidence identified.
Nature of the behaviour	✘	No further evidence identified.

## Discussion

### Principal findings

Midwives perceived a multitude of barriers and facilitators to carrying out HePPBes. Key barriers were requirements to perform an increasing amount of HePPBes on top of existing clinical work load, which impacted on the time available, midwives’ cognitive resources and the quality of relationships with pregnant women. Organisational issues such as a lack of continuity of care and difficulty accessing appropriate training were also identified. Key facilitators included midwives’ motivation to support pregnant women to address their health. Study 1 also highlighted strategies that midwives use to overcome the barriers they face in carrying out their HePPBes. Some findings were considered both barriers and facilitators as mixed views were expressed about whether certain health promotion topics should be addressed by other health professionals prior to pregnancy, women’s receptiveness to HePPBes during pregnancy and the social influence of midwives’ own health status.

### Strengths and limitations

The complimentary nature of the two presented studies is a strength. Study 1 provided detailed insight from a group of midwives working in a community setting which was supplemented in study 2 by free text commentary from a larger sample of midwives, employed within a variety of professional roles.

Limitations include the difficulty to specify target behaviours when simultaneously investigating multiple HePPBes for a variety of health promotion topics at the same time. The use of the TACT principle [16], and the image within the A4 prompt card provided midwives with a visual aid to remind them of the study focus during the interview. The sample size in study 1 was based on evidence-based guidelines [22], but is smaller than other qualitative TDF-based studies [23, 24]. In addition, the midwives who took part in study 1 were recruited from a single out-patient maternity clinic in Scotland and different and additional barriers and facilitators might have emerged within different contexts.

Study 2 used online recruitment which prevents checking that participating individuals fully met inclusion criteria. The current paper examined HePPBes at a general level but some of the barriers raised were health promotion topics specific (e.g. a lack of dietary services to refer women to). Future research could further explore similarities and differences of HePPBes for different health promotion topics.

### Relation to other studies

Limited evidence exists on the psychological factors associated with midwives HePPBes targeting women’s multiple health behaviours. Previously identified barriers to midwives undertaking HePPBes including a lack of time, resources and variability in training quality [6] were confirmed in the current study and therefore highlight a continued need for midwives to be provided with support. Uncertainty amongst midwives about their public health role [7, 8] was also demonstrated through the mixed views midwives expressed regarding whether all HePPBes should fall under the remit of the midwife. Midwives’ use of strategies to overcome the barriers they face in carrying out HePPBes has not been previously reported.

Examining multiple HePPBes increases the complexity of the behavioural influences identified and provides greater understanding of the influences on midwives HePPBes. The complexity of investigating multiple HePPBes is demonstrated by the higher number of barriers identified within the current study compared with studies which have used the TDF to explore midwives’ behaviours in relation to single health risk topics [11, 13, 14].

The TDF [10] provides an overview of the main psychological constructs explaining health behaviours. However, the theories that these constructs belong to are mainly used to explain single behaviours. Multiple behaviour change processes such as goal facilitation [25] and goal conflict [26] and transference [27] have not been captured by the TDF domain interview questions and therefore might have been missed by the current study.

### Possible mechanisms and implications

Barriers such as difficulty to access HePPBe-related training suggest a specific public health component in midwife training or after qualification may be useful. The finding that carrying out HePPBes can be taxing suggests that more support for midwives may be required. Policy makers and key stakeholders commissioning midwives’ continuous professional development opportunities could provide HePPBe support in multiple formats (e.g. through training, handheld materials or peer support).

Given the variations in the type of care that midwives provide, the pressure placed on maternity services by midwives attending training and the limited time that midwives would have to access support, developing handheld (or electronic) materials may be the most feasible option. For example, a leaflet containing examples of the strategies midwives use to carry out their HePPBes, that midwives could refer to during or outwith antenatal consultations, could capitalise on some of the HePPBe facilitators identified within this study.

### Unanswered questions and future research

The development of an intervention to support midwives in helping pregnant women address multiple health behaviours is necessary to maximise the effectiveness of public health interventions aimed at behaviour change during pregnancy. Future studies should translate the current findings into acceptable, scalable and effective interventions to support midwives to perform HePPBes.

## Conclusion

The findings suggest that despite high levels of motivation to carry out HePPBes, midwives perceive numerous barriers to carrying out these tasks in a timely and effective manner. Interventions that support midwives by addressing key barriers and facilitators to help pregnant women address their health behaviours are urgently needed.

## Additional files

Additional file 1: Study 1 Interview Topic guide. (DOCX 19 kb)

Additional file 2: Study 1 Prompt card. (DOCX 2283 kb)

Additional file 3: COREQ checklist. (DOCX 18 kb)

Additional file 4: Study 1 table of midwives view statements table. (DOCX 18 kb)

Additional file 5: Study 1 table of midwives HePPBe strategies. (DOCX 16 kb)

## Data Availability

The data that support the findings of this study are available from the corresponding author upon reasonable request.

## Abbreviations

COREQ: Consolidated criteria for reporting qualitative research
HePPBes: Health promotion practice behaviours
TDF: Theoretical domains framework
