# Supplementary material for: Investigating midwives’ barriers and facilitators to multiple health promotion practice behaviours: a qualitative study using the theoretical domains framework
Source: Implement Sci. 2019 Jun 18;14:64. doi: 10.1186/s13012-019-0913-3 (PMC6582467; doi:10.1186/s13012-019-0913-3)
Supplement: Supplementary file 1 — Study 1 Interview Topic guide. (DOCX 19 kb) [file 13012_2019_913_MOESM1_ESM.docx]

| **Additional file 1:**  **Study 1 interview topic guide** | |
| --- | --- |
|  | **Interview Topic Guide**  Participant ID: |
|  | *Today, I’m going to ask you some questions about your thoughts on the things you do to support pregnant women change their health behaviour, like smoking/drinking/substance abuse/diet and physical activity. There are no right or wrong answers and your responses will be anonymised. If you don’t want to answer a question, then please just say “I’d rather not answer that” and I’ll move onto the next one.*  *Do you have any questions before we begin?* |
|  | *To start us off, can you please tell me a bit about you and current role*  *What is your job title?*  *what band you are employed at:*  *where do you work?*  *and how many years’ experience have you got working as a midwife?*  *Are there any health behaviours that you are particularly involved in helping women address?* |
|  | *Ok, now that I have a bit more of an idea of your background, I’ll focus in on asking you about your views of what you do to help women change their health behaviour.* |
| **1** | ***Nature of the behaviours*** |
|  | *Can you tell me some of the things that you do to support pregnant women to change their health behaviour*? **(*Prompt: like smoking/drinking/substance abuse/diet and physical activity)***  *Are there any other things you do (to support pregnant women to change their health behaviour)?* |
| **2** | **Knowledge** |
|  | *Please tell me what you think that midwives are expected to do in order to try and help pregnant women change their health behaviour?*  *What is expected of you in regard to helping pregnant women change their health behaviour?* |
| **3** | **Skills** |
|  | *What skills do you need, as a midwife, to support pregnant women to change their health behaviour?*  *Do these skills differ for different health behaviours?*  *Do you think you have all of these skills?* ***(Prompt: Have you been trained? What did you think of the training? Did you need it?)*** |
| **4.** | **Social/ professional role and identity (self-standards)** |
|  | *Do you think supporting health behaviour change in pregnant women should be part of the role of a midwife?*  *Do you think the role of midwives in supporting pregnant women to change their health behaviour has changed over time?*  *If yes, how?* |
| **5.** | **Beliefs about capabilities (self-efficacy)** |
|  | *How confident are you that you can support pregnant women change their health behaviour?*  *Are there any health behaviours that you are especially confident about, or equally, not very confident about?* |
| **6.** | **Beliefs about consequences (anticipated outcomes/attitude)** |
|  | *What do you think are the benefits of helping pregnant women change their health behaviour? For you as a midwife/ the women?*  *What do you think are the bad things of helping pregnant women change their health behaviour? For you as a midwife/ the women?*  *Do you think the benefits outweigh the bad things?* |
| **7.** | **Memory, attention and decision processes** |
|  | *Is supporting pregnant women to change their health behaviour something that you usually do?*  *Are there other things that get in the way of you supporting pregnant women change their health behaviour?* |
| **8.** | **Motivation and goals (intention)** |
|  | *How important is helping pregnant women change their health behaviour in comparison to all the other things that you have to do?*  *Are there any health behaviours that you are particularly keen to help change?*  *Are there any health behaviours that you are not keen to change?* |
| **9.** | **Social influences (norms)** |
|  | *Do you think your colleagues help pregnant women change their health behaviour?*  *Do you sometimes talk with your colleagues about supporting pregnant women change their health behaviours?*  *What do you talk about? Do you support each other in this aspect of your job?* |
| **10.** | **Environmental context and resources (environmental constraints)** |
|  | *Tell me about the challenges of helping pregnant women change their health behaviour in your workplace?*  *Do you think you have enough resources to support pregnant women change their health behaviour? Time, materials, training, pathways, support and supervision?* |
| **11.** | **Emotion** |
|  | *How do you feel when trying to help pregnant women change their health behaviour? (Prompt e.g. feel emotionally)*  *Do you sometime avoid raising certain health behaviour topics with pregnant women because talking about this makes you feel a certain way, e.g. uncomfortable or awkward?* |
| **12.** | **Behavioural Regulation** |
|  | *Are there ways of working that encourage you to help pregnant women change their health behaviour?*  *Is there anything that could help encourage you to help women change their health behaviour?* |
|  | *That’s all my questions answered. Do you have anything you’d like to ask?*  *Thank you for taking part in the study. If you’d like to hear more about it then please contact Evelyn Frame or myself for a summary.* |
