# Supplementary material for: Investigating midwives’ barriers and facilitators to multiple health promotion practice behaviours: a qualitative study using the theoretical domains framework
Source: Implement Sci. 2019 Jun 18;14:64. doi: 10.1186/s13012-019-0913-3 (PMC6582467; doi:10.1186/s13012-019-0913-3)
Supplement: Supplementary file 2 — Study 1 Prompt card. (DOCX 2283 kb) [file 13012_2019_913_MOESM2_ESM.docx]

**Additional file 2: Study 1 prompt card**

All the things you do in a routine antenatal care consultation, including asking questions, to support pregnant woman change their health behaviours
