# Supplementary material for: Investigating midwives’ barriers and facilitators to multiple health promotion practice behaviours: a qualitative study using the theoretical domains framework
Source: Implement Sci. 2019 Jun 18;14:64. doi: 10.1186/s13012-019-0913-3 (PMC6582467; doi:10.1186/s13012-019-0913-3)
Supplement: Supplementary file 4 — Study 1 table of midwives view statements table. (DOCX 18 kb) [file 13012_2019_913_MOESM4_ESM.docx]

**Additional file 4: Study 1 table** **of key TDF domains, reasons for domain considered as key, midwives’ views and type of view**

| **Key TDF domains** | **Reason for domain being considered key** | **Midwives’ views (b= barrier and f= facilitator)** | **Generic or Behaviour specific**  **view** |
| --- | --- | --- | --- |
| Professional role and identity | Existence of conflicting beliefs | Carrying out HePPBes is part of my professional role (f)  Many of the HePPBes, expected of me, particularly those concerning weight management and/or obesity, could be undertaken by other health professionals prior to conception (b) | Generic and behaviour specific |
|  | High frequency of specific beliefs | Midwives role has evolved from providing traditional midwifery care (e.g. measuring the growth of the baby) to carrying out HePPBes (b) | Generic |
| Beliefs about consequences | Existence of conflicting beliefs | HePPBes, can develop my relationship with the woman in my care (f)  HePPBes, particularly those concerning weight management and/or obesity, can potentially damage my relationship with the woman in my care (b) | Generic and behaviour specific |
|  |  | Women are receptive to HePPBes at booking (f)  Women are not receptive to HePPBes at booking (b) | Generic |
|  | Indication of clear beliefs | Carrying out HePPBes can take up time and make me late for the next appointment (b) | Generic |
|  |  | HePPBes have the potential to have positive long-term benefits for the health of women and their baby (f) | Generic |
|  |  | I will feel rewarded if I see HePPBes improving the health of a woman and her baby (f) | Generic |
|  |  | If I carry out HePPBes effectively at the booking appointment, and the woman takes the information on board, it will make the rest of the pregnancy easier to manage (f) | Generic |
|  |  | The degree to which HePPBes can make a difference to a woman’s health in the short-term is specific to the individual behaviour (b) | Behaviour specific |
| Motivation and goals | High frequency of specific beliefs | I am motivated to carry out HePPBes to benefit the health of the woman and the baby (f) | Generic |
|  |  | HePPBes are important but are less of a priority than ensuring patient safety (b) | Generic |
| Memory, attention and decision processes | Indication of clear beliefs  High frequency of specific beliefs | The woman’s maternity notes prompt me to ensure I have carried out all my HePPBes (f) | Generic |
|  |  | If the woman specifically wanted to discuss a health topic related to HePPBes then this will cause me to focus on related HePPBes (f) | Generic |
|  |  | I focus on the HePPBes that concern the health topics that are most relevant to the woman (f) | Generic |
|  |  | My intuition helps me to make decisions about HePPBes (f) | Generic |
|  |  | The health of the woman at the antenatal appointment influences my decisions about HePPBes (b) | Generic |
| Environmental context and resources | Indication of clear beliefs | The current model of maternity care makes it more difficult to carry out HePPBes as there is less continuity of care (b) | Generic |
|  |  | The quality of HePPBe related training is variable and sometimes difficult to access (b) | Generic |
|  |  | There are too many HePPBes to carry out in too little time (b) | Generic |
|  |  | There are few dietary services I can refer women to (b) | Behaviour specific |
|  |  | The written materials, relating to HePPBes, I use with women are of high quality (f) | Generic |
|  |  | The questions in the woman’s hand-held maternity notes make some HePPBes difficult to perform (b) | Generic |
|  |  | If a woman is unwell at the booking appointment it is harder to carry out HePPBes (b) | Generic |
|  |  | Physical cues e.g. smell of smoke or teeth visibly in poor condition prompt me to carry out HePPBes (f) | Behaviour specific |
| Social influences | Existence of conflicting beliefs | The internet is a helpful influence in carrying out HePPBes (f)  The internet is an unhelpful influence in carrying out HePPBes (b) | Generic |
|  |  | Women are very honest when reporting their alcohol consumption (f)  Women are not honest when reporting their alcohol consumption (b) | Behaviour specific |
|  |  | My colleagues support me in carrying out HePPBes (f)  My colleagues do not support me in carrying out HePPBes (b) | Generic |
|  |  | Social and group norms can be helpful in normalising both my HePPBes and healthy behaviours (related to HePPBes) (f)  Social and group norms can be unhelpful in normalising obesity (b) | Generic and behaviour specific |
|  | High frequency of specific beliefs | A midwives’ own BMI can make HePPBes relating to weight management and/or obesity harder to carry out (b) | Behaviour specific |
| Emotion | Indication of clear beliefs | HePPBes can result in feeling positive feelings such as satisfaction (f) | Generic |
|  |  | Referring women to social work can be anxiety provoking (b) | Behaviour specific |
|  |  | It can be exhausting carrying out HePPBes amongst everything else I am required to do when providing antenatal care (b) | Generic |
| Behavioural regulation | High frequency of specific beliefs | I have specific strategies that I use to help me carry out HePPBes (f) | Generic |
| Nature of the behaviour | High frequency of specific beliefs | Undertaking HePPBes is a routine part of antenatal care (f) | Generic |
