# Supplementary material for: Investigating midwives’ barriers and facilitators to multiple health promotion practice behaviours: a qualitative study using the theoretical domains framework
Source: Implement Sci. 2019 Jun 18;14:64. doi: 10.1186/s13012-019-0913-3 (PMC6582467; doi:10.1186/s13012-019-0913-3)
Supplement: Supplementary file 5 — Study 1 table of midwives HePPBe strategies. (DOCX 16 kb) [file 13012_2019_913_MOESM5_ESM.docx]

**Additional file 5: Study 1 table of midwives HePPBe strategies**

| **Strategy** | **Quotation example** | **TDF domain coded in** |
| --- | --- | --- |
| Prioritisation (M5) | “S*ay I did three bookings yesterday one of them would have had none of these problems, one of them had a BMI was over 35 so that’s the one I concentrated on. Another one, yesterday, okay she was drinking, so that’s the one I concentrated on. Very rarely we focus on all of them with every person so, I would concentrate on the one that is relevant to that person”* | Behavioural regulation |
| Woman’s choice (M6) | “I*f the woman is worried about her weight, I’m happy to talk about it at every appointment, but if she’s not then I’m not gonna bring it up, same with alcohol”* |  |
| Brief interventions (M5) | *“I feel you can use a brief intervention and I know it is brought up around alcohol, but I use it for most things now”* |  |
| Making HePPBes into a conversation (M7) | *“We’re saying time is an issue but sometimes revisiting doesn’t take a whole lot of time, it’s just a conversation: “How you getting on with that?” “I know that was a wee bit of an issue for you last time and that you were struggling with that, but has it got any better?””* |  |
| Multi-task (M5&7) | *“You can be talking about their diet while you’re doing their blood pressure. You could be talking about their diet while you’re dipping their urine, while you’re feeling their tummy. You can do it in a conversation.”*  *“I have to say I do it as a multi task. I’ll be testing the urine while I’m asking about how they feel in pregnancy and have they had any sickness and how they’re getting on with eating and things like that. I’ll be multi-tasking the whole way.”* |  |
| Frame information as a positive  (M11) | *“If you can just frame it in such a way that makes it sound like a positive “this is what you can” do rather than “this is what you’ve been doing wrong” then you can maybe get round that.”* |  |
| Dipping  (M9) | *“I think it's about dipping in to different things not just the first appointment but about mentioning later on “tell me about your husband's alcohol intake, how is that impacting?"* |  |
| Chipping  (M7) | *“So what bit for you do we need to look at?” because there’s very few people that need absolutely, well some of them do need absolutely everything, but if they do it’s about chipping away at it. I think you have to think let’s look at this wee bit by bit.*  *“Next appointment we might have to chip away at something else”* |  |
| Information provision  (M5 & 11) | *“I’m kind of the opinion I give people the information and make sure they know that if they have any questions they can ask.”*  *“I try and identify areas that they may need more information about.”* |  |
| Prompting from maternity notes  (M10&11) | *“It’s there in front of you and you know you’re to discuss these things”*  *“I usually always have a wee flick through the notes at the beginning just to check if there’s any kind of outstanding issues or anything to be aware of”* | Behavioural regulation and Nature of the behaviour |
| Use of materials  (M3) | *“It would just be generally going through the “Ready, Steady, Baby” book that they're given”* |  |
| Referral  (M10) | *“A lot of this time you’re referring on to other services like smoking cessation. If there’s any history of domestic abuse you’re referring on to other services. If the woman is overweight, you are referring on to services related to that like exercise classes.”* |  |
